# Whole-mount specimens in the analysis of en bloc samples obtained from revisions of resurfacing hip implants: A report of 4 early failures

**DOI:** 10.3109/17453674.2010.480934

**Published:** 2010-05-21

**Authors:** Ioannis Stogiannidis, Timo Puolakka, Jorma Pajamäki, Teemu Moilanen, Yrjö T Konttinen

**Affiliations:** ^1^Department of Orthopaedic Surgery, Coxa Hospital for Joint Replacement, Tampere; ^2^Department of Medicine, Institute of Clinical Medicine, Helsinki University Central Hospital, HelsinkiFinland

## Abstract

**Background:**

Modern metal-on-metal hip resurfacing implants are being increasingly used for young and active patients, although the long-term outcome and failure mechanisms of these implants are still unknown. In this consecutive revision case series, early failures of femoral implants (at < 4 years) were studied.

**Methods:**

3 revisions were done due to a fracture of the femoral neck and 1 due to loosening and varus position of the femoral component. Femoral heads were removed en bloc 2–46 months after the primary operation, embedded in methylmethacrylate, sectioned, stained, and analyzed as whole-mount specimens in 4 55–62-year-old patients with osteoarthritis.

**Results:**

Histopathology was characterized by new but also partly healed trabecular microfractures, bone demineralization, cysts, metallosis, and abnormal formation of new woven bone. All samples displayed signs of notching, osteoporosis, and aseptic necrosis, which seemed to have been the main reason for the subsequent development and symptoms of the patients and revision operations of the hips.

**Interpretation:**

Based on these early revision cases, it appears that aseptic necrosis is a common cause of early loosening of resurfacing hip implants.

## Introduction

Metal-on-metal hip resurfacing preserves the size of the femoral head, which improves stability of the joint and prevents hip implant dislocations. Resurfacing preserves proximal femoral bone so that revision operation of this component is easier than after a conventional hip replacement. The renaissance of metal-on-metal articulations for total hip arthroplasty is based on the use of improved metallic biomaterials, improved implant design, and improved production methods (Konttinen et al. 2008). The early results are encouraging, as complications commonly seen in the 1970s and 1980s, such as early implant loosening (Little et al. 2005, Shimmin et al. 2005), have been rare (Grigoris et al. 2005). Some studies indicate survival rates of over 97% with follow-up from 2 to 8 years (Amstutz et al. 2004, Daniel et al. 2004). Sometimes resurfacing hip implants have to be revised relatively early during the 2 first years after implantation (www.jru.orthop.gu.se, Annual Report 2007, page 27), and here we report 4 such early failures (Table). As it has been reported that resurfacing hip implants lead to release of high concentrations of metal ions rather than formation of foreign bodies, it was hypothesized that revised cases would be characterized by chronic mononuclear cell infiltrates composed mostly of lymphocytes (representing delayed-type hypersensitivity reaction) rather than monocyte/macrophages (representing chronic foreign body reaction) (Willert et al. 2005).

## Patients and methods

### Case 1

**Figure 1.**
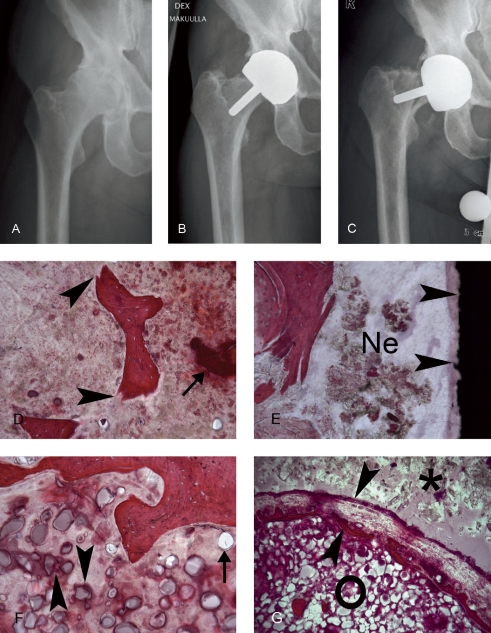
Radiographs of the right hip of case 1, a male patient with end-stage osteoarthritis. A. A preoperative anteroposterior radiograph showing good bone quality. B. A postoperative anteroposterior radiograph showing that the BHR implant is well-positioned. C. 46 months postoperatively, this patient fell on stairs. Radiographs showed a femoral fracture. D. A fractured bone trabecula (arrowheads) in an area adjoining necrotic bone (arrow). E. Implant-host tissue interface with some necrotic tissue (Ne) close to the black implant surface (arrows). F. Trabecular bone with marrow space, which contains phagocytozed (arrowheads) and extracellular (arrow) cement particles. G. Such cement particles (o) were often seen close to the host-cement interface (*), which in this section is already separated by an implant capsule (arrowheads).

A 59-year-old male with osteoarthritis of his right hip underwent hip resurfacing arthroplasty and received a BHR implant. He fell x months later on stairs, receiving a fracture of the femoral neck (Figure 1, panels A–C).

### Case 2

**Figure 2.**
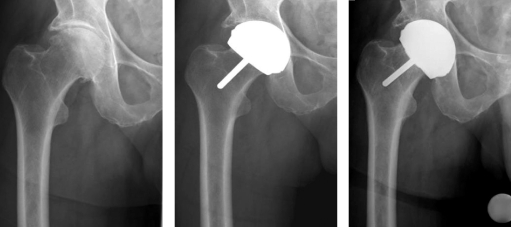
Radiographs of case 2, a male patient with osteoarthritis in the right hip. A. A preoperative anteroposterior radiograph showing good bone quality. B. A postoperative anteroposterior radiograph showing that the implant is well-positioned. C. 7 months postoperatively, the patient developed pain in his hip always after intensive physical activity. Radiographs showed that the femoral component had now turned to a varus position.

A 62-year-old male with osteoarthritis in his right hip received a resurfacing Durom hip implant. He suffered from a cardiovascular disease and asthma, had osteoarthritis of the hip, and received a Durom implant but at 7 months developed pain after intensive physical activity. Radiographs showed that the femoral component had turned into varus (Figure 2, panels A–C). There was no radiographically apparent evidence of avascular osteonecrosis because the necrotic areas were hidden inside the metal shell of the resurfacing implant (Figure 2); necrosis was first revealed at revision. He was 1 of the 2 patients in this series who used glucocorticosteroids for asthma.

### Case 3

A 55-year-old female with osteoarthritis of both hips underwent a bilateral hip arthroplasty, receiving a resurfacing ASR implant on the left side, whereas her right side was treated with a conventional stemmed prosthesis. At 2 months she developed pain in her left hip, fell on stairs, and sustained a subtrochanteric fracture.

### Case 4

A 58-year-old male with osteoarthritis of both hips underwent a bilateral hip resurfacing arthroplasty, performed using BHR implants. He suffered from a cardiovascular disease and asthma. At 20 months he fell from a horse, pain continued, and radiographs showed a femoral neck fracture.

### Radiographic analysis

The radiographs of the patients were reviewed for evidence of possible mechanical stress factors and their consequences, which could predispose to loosening or fracture, including the position of the implants, notching of the femoral neck, and avascular necrosis.

### Histological analysis of the revised resurfacing implant-bone composite samples

An extended posterolateral approach was used in all procedures. At the revision operation, the femoral component together with the femoral head and neck bone was resected en bloc and immediately placed in 10% formalin for 4 weeks. No acetabular components were removed. After fixation, the bone-metal composite specimens were dehydrated in a graded ethanol series, cleared in xylene, and mounted in methylmethacrylate. Slices 2–3 mm thick were cut using a diamond saw and ground to 80-μm-thick sections. Sections were stained at 22°C.

Methylmethacrylate was first dissolved in methoxyethyl acetate for 3 × 10 min and sections were partially rehydrated in 96%, 70%, and 40% ethanol for 4 min in each, before staining in Harris hematoxylin for 8 min. The sections were blued in tap water for 10 min and stained for 5 min in eosin. The slides were dried in absorbent paper before dehydration in ethanol, clearing in xylene, and embedding in Mountex (Histolab, Gothenburg, Sweden). The stained whole-mount specimen sections were inspected using light microscopy and photographed.

## Results

### Case 1

**Figure 3.**
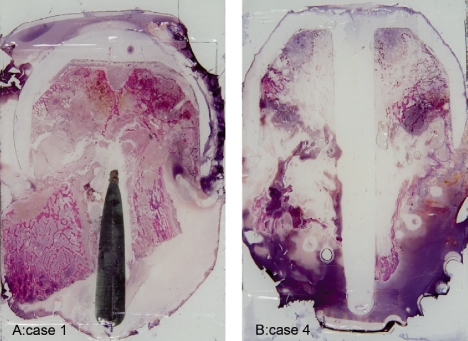
Macroscopic sections of 2 cases, which display femoral neck fractures and aseptic necrosis; case 1 (panel A) and case 4 (panel B).

The head of the femur had undergone necrosis and had lost its normal trabecular bone architecture as detailed in Figure 1 (panels D–G) and Figure 3 (panel A).

### Case 2

Macroscopic analysis of the implant-bone composite specimen removed at the revision operation disclosed an apparently empty defect area in the middle of the section of the specimen, which in radiographs had been concealed inside the metal shell of the femoral component. In addition, neck melting was seen in the area where the rim of the femoral shell was in contact with bone. A large and already macroscopically evident cystic bone defect in the femoral neck was bordered by thin osteoporotic bone trabeculae. In the surrounding bone, numerous microfractures of bone trabeculae with fibrotic or bony callus (signs of ongoing healing) were seen, indicating that these were generated in vivo and were not in vitro artefacts caused by sample processing. They were sometimes surrounded by a mild inflammatory reaction and bone marrow fat. Clear metallosis was often seen, and most but not quite all of the polymethylmethacrylate had been dissolved as a result of tissue processing—and had left voids. The peri-implant trabecular bone and marrow did not contain any implant capsule or chronic mononuclear macrophage-rich or lymphocyte-rich inflammatory cell infiltrates. Finally, the implant stem (the metallic pin used for fixation of the femoral component) had been detached from the photographed section during grinding, but the void after it was still clearly discernable due to its central medullary location and geometric contours. The margins of the stem canal contained some remains of bone cement (polymethylmethacrylate), which in this case had perhaps been protected from dissolution by bone trabeculae. At the same time, its presence indicates that the attempts to prevent penetration of the low viscosity cement into the medullary canal had not quite been successful in this case.

### Case 3

The surface of the head of the femur already consisted of a mixture of cement and amorphous necrotic bone, which no longer contained discernable cell nuclei. In some areas this mixture formed finger-like extensions, which intruded towards the center of the head. The bony trabeculae at the margins of these projections were eroded and partly reorganized, which was in contrast to bone in the deeper parts of the femoral head; these contained still intact and vital bone trabeculae. This healthier zone bordered on the neck of the femur, which again contained small, resorbing, and necrotic bone trabeculae (not shown). Remnants of cement showed that the cement layer was thick but fragmented in this patient.

### Case 4

**Figure 4.**
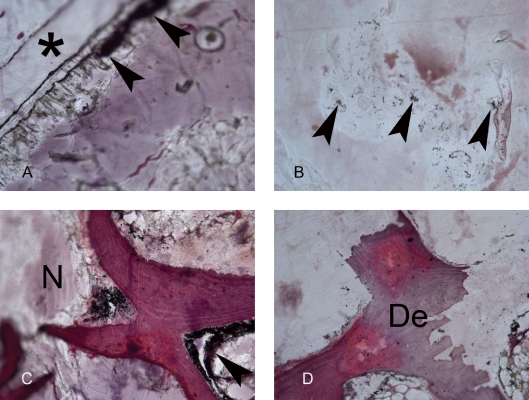
Histology of case 4. A. The implant, which covered the femoral head, became detached (*) during cutting and grinding. Bone tissue below it was infiltrated with aggregates of small dark metal wear-debris particles (metallosis, arrowheads). B. This was also seen in deep tissues in the femoral neck that had been in contact with the implant stem; some of the metal particles have been marked with arrowheads. C. Bone trabeculae between a necrotic area (to the left, marked with N) and healthier bone (to the right). Some metallosis (arrowheads) can be seen in close association with the bone trabeculae in this view. D. In many areas, trabeculae were not only broken but also apparently undergoing demineralization (De), as in this image.

The femoral neck fracture had caused an extensive aseptic necrosis and bone defects (or vice versa), as shown in Figure 3 (panel B) and Figure 4 (panels A–D).

**Figure 5.**
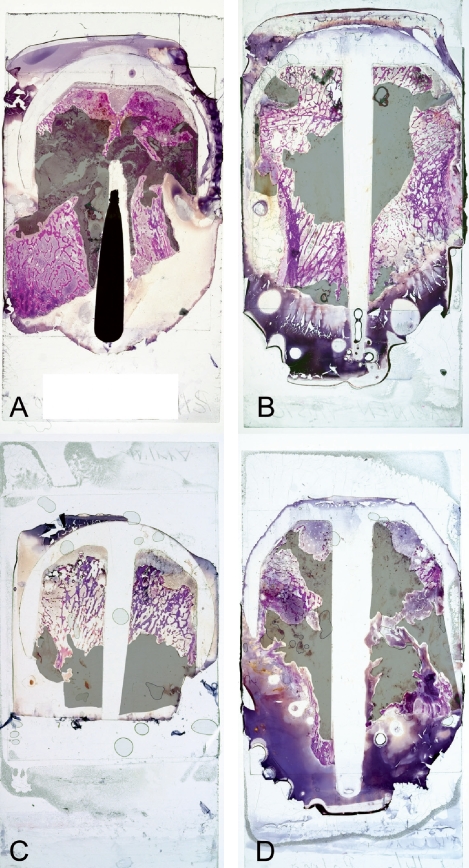
Necrotic areas of the femoral heads in the 4 cases studied have been overlayed with transparent gray masks to show their extent: case 1 (panel A), case 2 (panel B), case 3 (panel C), and case 4 (panel D).Table 1. Summary of the 4 osteoarthritis patients at the time of the primary operation, in whom the femoral head and the resurfacing implant were removed for analysis

An illustration of the necrotic areas in all 4 cases is presented in Figure 5.

## Discussion

In our study neck melting or notching, osteoporosis of the head and neck of the femur, avascular caput necrosis and femoral neck fractures were seen. Pedestal formation and radio-opaque lines at the tip of the implant stem adjacent to a radiolucent area were not seen (Ong et al. 2006). We hypothesized a priori that these cases would be characterized by a lymphocyte-mediated, delayed-type hypersensitivity reaction to metallic ions acting as haptens, which bind to endogenous proteins and change “self” to “altered self” or “non-self”. This stimulates the host immune response, which is characterized by high endothelial cell venules, fibrin exudation, diffuse perivascular infiltrates of T and B lymphocytes and plasma cells, accumulation of macrophages with drop-like inclusions, and infiltrates of eosinophilic granulocytes and necrosis (MacDonald 2004, Willert et al. 2005). However, we did not observe such features in spite of the fact that these tissues were clearly affected by heavy metallosis. This lack of any hypersensitivity reaction could have 3 main reasons. Firstly, these revisions were all relatively early ones, so it might be that the host immune system had not yet had time to become sensitized and to develop a histopathologically evident delayed-type hypersensitivity reaction. Secondly, the third-generation resurfacing hip implants are made of cobalt-chromium; cytotoxicity of the chromium released from the implant might protect against such host responses by killing the potentially reactive cells. Thirdly, the reason for the lack of such a response could be trivial, because we only analyzed only 4 cases. Metal-to-metal pairs are wear-resistant and produce less wear debris than conventional metal-to-polyethylene gliding pairs, which reduces the risk of chronic foreign body inflammation.

According to our study, loss of bone and weakening of its microarchitecture in the proximal parts of the femur in patients with resurfacing implants can have not only one but several explanations. Our cases all showed impingement at the superolateral neck area, indicating that the metal shell of the resurfacing implant impinges upon the adjoining bone. When the stem of the resurfacing Birmingham hip was not fixed (or had become loose), resorption was limited to the superolateral region (Ong et al. 2006). The superolateral edge of the implant shell appears to be a high contact stress point, perhaps due in part to the load-bearing nature of the implant and as a result of the static and cyclic weight bearing during walking. This naturally leads to local weakening of the femoral neck.

Resurfacing hip implants have been marketed in part by advertising their physiological stress-distributing properties. This has been documented in the femoral shaft area, which, in patients with resurfacing hip implants, is subjected to relatively normal weight bearing cyclic stress (Kröger et al. 1998, Kishida et al. 2004, Harty et al. 2005). In contrast, in conventional total hip replacement the thick and strong metallic femoral stem carries most of the load, so that stress shielding ensues—leading to demineralization of the peri-implant bone, which predisposes to peri-implant bone fractures. According to our results, this advantage is combined with a negative effect in the femoral collum area, which was characterized by thin and sparse bone trabeculae indicating stress shielding and bone loss or weakening in this anatomical site. This was also evident from multiple microfractures of the bone trabeculae, in different areas of the whole-mount samples and at different stages of healing. While these changes can be minor, excessive remodeling and satress may lead to femoral neck fracture (Shimmin and Back 2005, Amstutz et al. 2007).

Our cases were characterized by large bone cysts or avascular bone necrosis of the head and neck of the femur at various stages of resorption. These defects might arise as a result of damage to collaterals of the circumflex femoral artery or other arteries, leading to platelet adhesion, aggregation, and thrombosis. Such vascular damage might be caused by the implantation procedure, including the use of guide pin, reamer, bone cement, and hammering, or by the implant shell—and particularly by its intramedullary stem at the time of insertion, or perhaps later if it is yielding and turning to a varus malposition. It has been suggested that the posterior surgical approach in particular destroys the important extraosseous blood supply to the femoral head (Freeman 1978, Mont et al. 2005, Beaulé et al. 2006). However, positron emission tomography (PET) suggests that an intraosseous collateral circulation develops in osteoarthritis, protecting the femoral head (McMahon et al. 2006). The presence of bone cysts and avascular necrosis of the bone heads in our cases clearly demonstrates that, at least in these patients, the collateral blood circulation was insufficient. Furthermore, due to vascular anomalies, some patients may be more vulnerable to such vascular damage and its consequences than others. Finally, if the weakened femoral neck fractures, this may cause additional, secondary damage to the circulation of the femoral head—as was probably the situation in 3 of our 4 cases. The signs and symptoms of aseptic necrosis and early loosening probably develop slowly over an extended period of time, although the symptoms can be aggravated by trauma. Loosening of a resurfacing hip implant is not an acute surgical catastrophe, and is therefore revised in an elective procedure. Some of the aseptic necrosis may therefore be secondary, due to the fracture, although one also has to consider that the fracture itself might very well be a pathological one.

Our observations suggest that the cement layer is probably fragmented relatively often, and that it does not cover the whole of the dead space between implant and bone. This may impair fixation and increase the biomechanical stresses and strains. As the cement mantle was several millimeters thick in 2 cases, it is possible that exothermic polymerization of the cement provides an additional mechanism of bone damage (Little et al. 2008). When low-viscosity Simplex cement is used, the implant is driven into its place by hammering, which may cause some impact damage to the bone and blood vessels.

**Table:** Summary of the 4 osteoarthritis patients at the time of the primary operation, their failure mode and months to revision in whom the femoral head and the resurfacing implant were removed for analysis

Case no.	Age/Gender	Implant	Mode of failure	Months to revision	Side/Bilateral	Cement type/Viscosity
1	59/M	BHR 46-mm head, 52-mm cup	Fracture of the femoral neck	46	Right	Simplex/Low viscosity
2	62/M	Durom 54-mm head, 60-mm cup	Loosening of the femoral component	7	Right	Simplex/Low viscosity
3	55/F	ASR 40-mm head, 45-mm cup	Fracture of the femoral neck	2	Left/Bilateral	Palacos/High viscosity
4	58/M	BHR 50-mm head, 56-mm cup	Fracture of the femoral neck	24	Right/Bilateral	Simplex/Low viscosity

F: female; M: male; BHR: Birmingham Hip Resurfacing implant (Smith and Nephew, Memphis, TE), Durom (Zimmer, Winterthur, Switzerland); ASR = Articular Surface Replacement (DePuy, Leeds, UK).
